# Supplementary material for: The Role of Marek’s Disease Virus UL12 and UL29 in DNA Recombination and the Virus Lifecycle
Source: Viruses. 2019 Jan 28;11(2):111. doi: 10.3390/v11020111 (PMC6409567; doi:10.3390/v11020111)
Supplement: Supplementary file 1 [file viruses-11-00111-s001.pdf]

viruses

MDPI

Supplementary Materials:

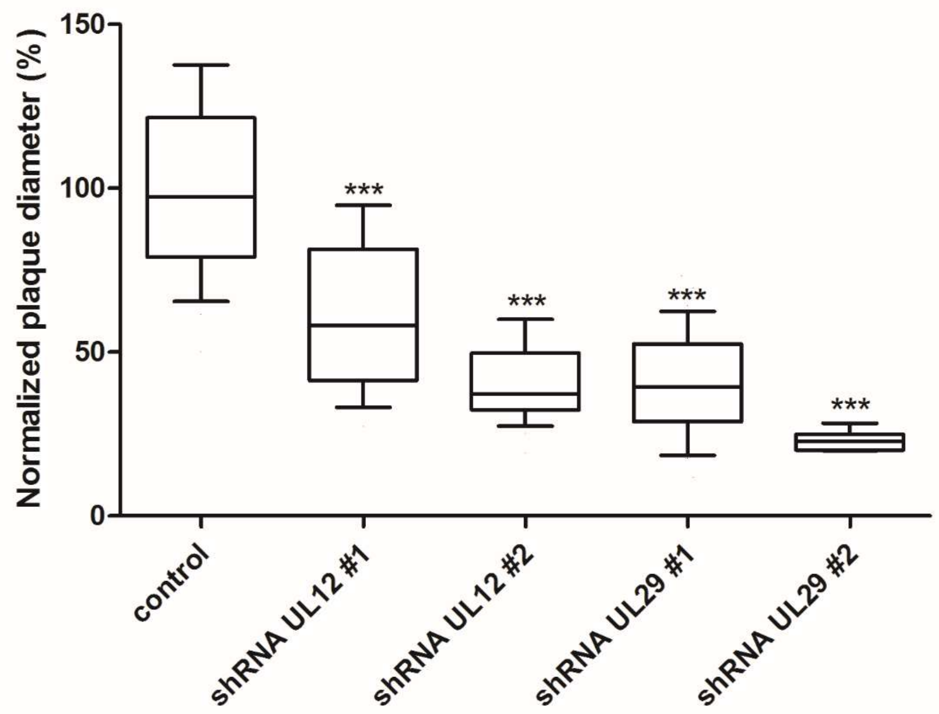

Figure S1. Validation of the UL12 and UL29 shRNA constructs. CR cells with or without shRNAs against UL12 or UL29 were infected with MDV and infected cells cultured. Plaque sizes were measured six days post infection. The average plaque diameters are shown as a box plot (normalized to wild type, n=50). Statistical differences in plaque diameters were determined using one-way ANOVA (***, P<0.0001).

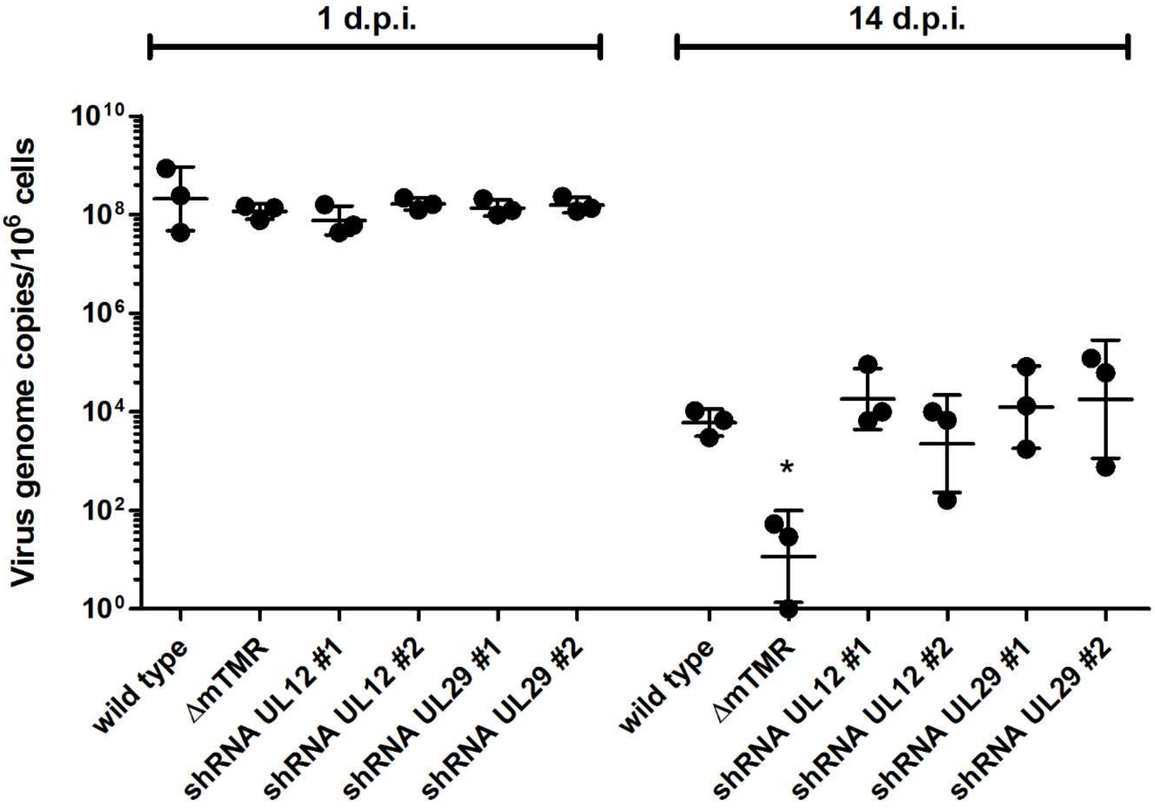

Figure S2. Role of UL12 and UL29 in MDV integration. CU91 T cells with or without shRNAs against UL12 or UL29 were infected with MDV. Samples were taken for qPCR analysis on day 1 and day 14. Displayed are the mean levels of virus genome copies per million cells (bar) ±SD from three independent experiments (each experiment is depicted by a single dot). Statistics were performed using the Kruskal-Wallis test (**P<0,01).

Table S1. Oligonucleotide sequences used in this study

Construct name/ qPCR		Sequence (5' → 3')
vUL12 mut1	For	CTCTTTCGTTTTCGGGACTCATCTTTGCAAGATTTGGAGAGTGGTTACTAGGTTCCTAGTTCCATATAGGGATAACAGGGTAATCGATTT
	Rev	CAGTAGCAAGAACGGCTGATTTGTTGTTATGGAACTAGGAACCTAGTAACCACTCTCCAAATCTTGCGCCAGTGTTACAACCAATTAACC
vUL12 rev1	For	TTCGTTTTCGGGACTCATCTTTGCAAGATTTGGAGAGTGGAAACTTGGTTCCTAGTTCCATAACAAGCCAGTGTTACAACCAATTA
	Rev	TAGCAAGAACGGCTGATTTGTTGTTATGGAACTAGGAACCAAGTTTCCACTCTCCAAATCTTGCAATAGGGATAACAGGGTAATCG
vUL12 mut2	For	GTGCCACAATTCACTGTTCGCTTGTAGTCTAGTTAACGATTGAGCTCATTTGCACCAGCGAGAAGTAGGGATAACAGGGTAATCGATTT
	Rev	GGTTAATTGGTTGTAACACTGGCTAGTCTAGTTAACGATTGAGCTCATTTGCACCAGCGAGAAGCATGAATTGATGGTGACAAATTAT
vUL12 rev2	For	TGCCACAATTCACTGTTCGCTTGTAGTCTAGTTAACGATTCCACCATTTTGCACCAGCGAGATAGGGATAACAGGGTAATCGATTT
	Rev	TAATTTGTCACCATCAATTCATGCTTCTCGCTGGTGCAAAATGGTGGAATCGTTAACTAGACGCCAGTGTTACAACCAATTAACC
vUL29 mut1	For	GCCTATCGGTCCGCCGCAAAGTTTCACGCTTTTTCCTACAAGCTTCTAAATGTTGTTGTTAGGGATAACAGGGTAATCGATTT
	Rev	GGTTAATTGGTTGTAACACTGGCCTTTTTCCTACAAGCTTCTAAATGTTGTTGTTGGATAGTTGACAGAATCTAGTGCGAGAG
vUL29 rev1	For	AGCCTATCGGTCCGCCGCAAAGTTTCACGCTTTTTCCTACACCATCCATAATGTTGTTGTTGGATAGGGATAACAGGGTAATCGATTT
	Rev	CTCTCGCACTAGATTCTGTCAACTATCCAACAACAACATTATGGATGGTGTAGGAAAAAGCGTGGCCAGTGTTACAACCAATTAACC
Sequencing of vUL12 mut1/rev1	For	TCGGACGGAAGATTGGCT
	Rev	CCACAGAGCCGAGTTTGATAA
Sequencing of vUL12 mut2/rev2	For	CACTGGGATAGTTCACTTCTCATTTC
	Rev	TAAACATTTTTCCCTATTTAAACAACCTGT
Sequencing of vUL29 mut1/rev1	For	ATAAATATATGACCTCTTTAAATAATTCGG
	Rev	TATGAGTTCCCTTTTGTTTTACATCTTT

ICP4	For	CGTGTTTTCCGGCATGTG
	Rev	TCCCATACCAATCCTCATCCA
	Probe	FAM-CCCCCACCAGGTGCAGGCA-TAM
iNOS	For	GAGTGGTTTAAGGAGTTGGATCTGA
	Rev	TTCCAGACCTCCCACCTCAA
	Probe	FAM-CTCTGCCTGCTGTTGCCAACATGC-TAM
UL12 expression plasmid	For	AGCGTTTAAACTTAAGGCCACCATGGAACTAGGAACCAAGTTTCC
	Rev	CCGAGCTCGGTACCTTAAATACGACACTGCTTGG
ICP8 low copy plasmid – CMV insertion	For	CAAAGTTTCACGCTTTTTCCTACACCATCCATAATGTTGTTGCACCGTACACGCCTACCG
	Rev	GTTATCTAGGAAGCTGATGCGGCCGGCCCTTAATTAACCATGTACGGGCCAGATATACGC
ICP8 low copy plasmid – BGH insertion	For	TCGTATAGCATACATTATACGAAGTTATCTAGCAGATCTGTTTGTCTTCCCAATCCTCCC
	Rev	CCGAGAAGCGCGTACCAGTGTTATCTGTAGATATGTTGTAAAGCATGCATCTAGAGGGCC
Sequencing of ICP8 low copy plasmid	For 1	ACCCCCACTGCCCAAAC
	Rev 1	GCGAGTTTACGTGCATGGAT
	For 2	AATGCCTGTCAAGGGCAAGT
	Rev 2	GAGTCCGAATGGTGCTATCC
